# Alternative Splicing as a Regulator of Early Plant Development

**DOI:** 10.3389/fpls.2018.01174

**Published:** 2018-08-15

**Authors:** Dóra Szakonyi, Paula Duque

**Affiliations:** Instituto Gulbenkian de Ciência, Oeiras, Portugal

**Keywords:** alternative splicing, early seedling development, embryogenesis, photomorphogenesis, seed dormancy, seed maturation, seed germination, splicing factors

## Abstract

Most plant genes are interrupted by introns and the corresponding transcripts need to undergo pre-mRNA splicing to remove these intervening sequences. Alternative splicing (AS) is an important posttranscriptional process that creates multiple mRNA variants from a single pre-mRNA molecule, thereby enhancing the coding and regulatory potential of genomes. In plants, this mechanism has been implicated in the response to environmental cues, including abiotic and biotic stresses, in the regulation of key developmental processes such as flowering, and in circadian timekeeping. The early plant development steps – from embryo formation and seed germination to skoto- and photomorphogenesis – are critical to both execute the correct body plan and initiate a new reproductive cycle. We review here the available evidence for the involvement of AS and various splicing factors in the initial stages of plant development, while highlighting recent findings as well as potential future challenges.

## mRNA Processing and Alternative Splicing

**FIGURE 1:**
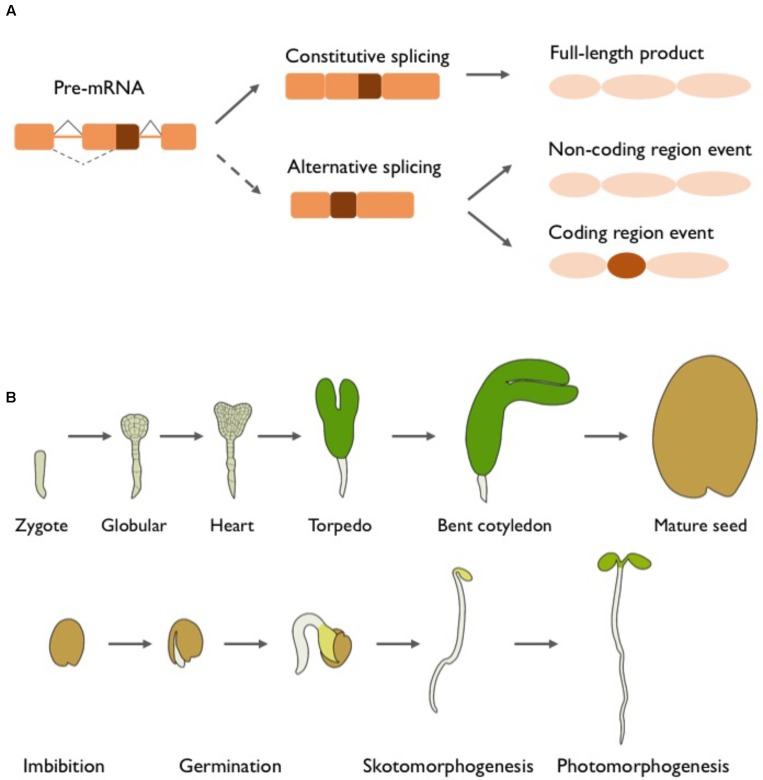
Alternative splicing and early plant development. **(A)** Constitutive and alternative splicing. Nascent multi exonic mRNAs need to undergo pre-mRNA splicing. Constitutive splicing removes the non-coding introns, producing a mature mRNA that encodes the full-length protein or transcript with biological functions. The same pre-mRNA molecule can undergo alternative splicing (AS) and produce different transcript variants. AS events occurring in non-coding sequences often impact gene expression, but will result in a protein identical to the full-length isoform. In numerous cases, coding regions are affected by AS, thus originating markedly different mRNAs and potentially distinct proteins that can vary in virtually all functional aspects. **(B)** Embryogenesis, seed maturation and germination, and early seedling development. After fertilization, the zygote undergoes a rapid succession of highly coordinated cell divisions to form globular stage embryos, which show establishment of the apical–basal axis and a first distinction between outer and inner cells. The embryonic cells further differentiate during the heart stage, when many of the basic cell types (provasculature, endodermis, cortex, and protoderm) and organ primordia (cotyledons, hypocotyl, and primary root) are formed and a bilateral body pattern appears. Expanding cotyledons give the embryo a torpedo shape, and the formation of the shoot and root apical meristems is completed. The next steps involve further cell growth and divisions until the embryo reaches its final shape and size. Seed maturation is completed with the accumulation of reserves and the establishment of desiccation tolerance and seed dormancy. Dry seeds are released from dormancy in response to a combination of environmental cues and internal signals. After water uptake, key biochemical and molecular processes are restored, followed by the rupture of the seed coat and emergence of the radicle, marking the completion of germination. Under darkness, the buried seedling undergoes skotomorphogenesis characterized by a short root, elongated hypocotyl, apical hook and absence of photosynthetic pigments. Upon light exposure, photomorphogenesis is activated leading to inhibition of hypocotyl elongation, opening and expansion of the cotyledons, and initiation of photosynthesis after chloroplast maturation.

Accurate processing of precursor mRNAs (pre-mRNAs) is a major step in gene expression crucial for performing everyday housekeeping functions, executing developmental programs, and responding to intrinsic and environmental cues. It involves modification steps to remove non-coding sequences as well as add the cap and the poly(A) tail to the 5′ and 3′ ends of the mRNA, respectively (reviewed in Proudfoot, 2011; Shi and Manley, 2015; Ramanathan et al., 2016). Pre-mRNA splicing, the excision of introns followed by joining of exons, is catalyzed by the spliceosome, a large ribonucleoprotein complex. The spliceosomal subunits assemble at conserved nucleotides at the exon-intron boundaries also known as the 5′ (or donor) and 3′ (or acceptor) splice sites (SS), the branch point and the polypyrimidine tract. In addition to the core spliceosomal components, many RNA-binding proteins play key roles in mRNA processing, SS selection and splicing (reviewed in Meyer et al., 2015). In higher eukaryotes, intron-containing genes frequently give rise to multiple mRNAs through alternative splicing (AS) (**Figure 1A**), during which differential recognition of SS can lead to intron retention, exon skipping and/or alternative 5′/3′SS selection. AS can significantly enhance a genome’s coding capacity by producing protein variants with altered function. It also often affects mRNA stability by introducing premature stop codons in the coding sequence, thus targeting these transcripts to degradation by nonsense-mediated decay (NMD). Furthermore, AS can modify gene expression by modulating transcription elongation and/or translation efficiency (reviewed in Reddy et al., 2013; Laloum et al., 2017). It is hence not surprising that AS fulfills important biological functions. In plants, it has been found to control key processes like the circadian clock or flowering time as well as the response to environmental cues, including abiotic stress or pathogen attack (reviewed in Staiger and Brown, 2013; Yang et al., 2014; Laloum et al., 2017; Shang et al., 2017).

## Early Plant Development

The first stages of a plant’s life are essential to establish the basic body pattern, develop different tissue types and initiate a new reproductive cycle (**Figure 1B**). Sexual reproduction of land plants involves the alternation of haploid and diploid stages. Angiosperms have a dominant diploid sporophyte and a relatively short haploid phase consisting of a few microscopic cells. Seeds are produced by double fertilization. One sperm cell fuses with the egg cell to form the diploid embryo, while a second sperm cell fertilizes the diploid central cell to give rise to the endosperm (reviewed in Raghavan, 2003; Berger et al., 2008). During embryogenesis, the one-cell zygote undergoes a tightly regulated developmental program to form a mature embryo. In dicots such as *Arabidopsis thaliana* (arabidopsis), this process includes distinct morphological stages, called globular, heart, torpedo, and bent cotyledon, leading to the establishment of the basic body plan and main tissue/organ initials including the shoot and root apical meristems (reviewed in Palovaara et al., 2016). Embryo morphogenesis is followed by seed maturation, which involves the accumulation of reserves, acquisition of desiccation tolerance, reduction of metabolic activities and induction of dormancy to enable survival of the embryo until favorable environmental conditions allow germination (reviewed in Graeber et al., 2012). Fresh seeds usually show high dormancy that gradually decreases over time in a process called after-ripening. The release from dormancy depends on environmental factors (e.g., light quality, day length, temperature, water availability, exposure to cold) and internal regulators (e.g., hormones, regulatory proteins, chromatin status) (reviewed in Kucera et al., 2005; Nee et al., 2017). Germination starts with water uptake (imbibition) and rapid expansion of the embryo, leading to rupture of the seed coat and emergence of the radicle. Seedlings growing in the dark display skotomorphogenic development (etiolated growth), characterized by elongated hypocotyls, apical hook, pale cotyledons and short roots. When exposed to light, the seedling undergoes photomorphogenesis to activate vegetative growth, displaying shorter and thicker hypocotyls as well as green and expanded cotyledons (reviewed in Wu, 2014). Hormones are important regulators of early plant development. Embryo formation is governed by auxins and cytokinins, while abscisic acid (ABA) is important for the completion of seed maturation and building up dormancy. ABA is also the major inhibitor of seed germination, with its effect being counteracted by gibberellic acid, ethylene, and brassinosteroids (reviewed in Palovaara et al., 2016).

## Global Alternative Splicing Changes During Early Plant Development

Next-generation sequencing has revolutionized transcriptomic studies. The latest RNA-seq data gathered in higher plants showed that traditional approaches largely underestimated the proportion of genes undergoing AS. Current assessments indicate that up to 70% of plant multiexon genes generate more than one transcript via this mechanism, with intron retention representing the predominant mode of AS (Lu et al., 2010; Zhang et al., 2010; Marquez et al., 2012; Shen et al., 2014; Thatcher et al., 2014; Chamala et al., 2015; Sun and Xiao, 2015; Iniguez et al., 2017; Zhang et al., 2017). In fact, increased sequencing coverage revealed a large number of non-annotated AS events and splice variants (Marquez et al., 2012; Zhang et al., 2017). Most of the plant AS events map to coding regions, thereby altering protein sequence and potentially function or compromising mRNA stability. Indeed, a significant proportion of intron-containing genes are potentially regulated by NMD (Zhang et al., 2010; Kalyna et al., 2012; Drechsel et al., 2013). Although thousands of alternatively spliced mRNAs are detected in genome-wide analyses, detailed genetic and molecular studies will be required to identify functionally relevant AS events.

Numerous plant large-scale studies have focused on gene expression and AS patterns in different tissues and during development, identifying many novel organ- or stage-specific mRNAs with dynamic expression changes and a stage-dependent switch in isoform dominance for many genes (Zhang et al., 2010; Thatcher et al., 2014; Klepikova et al., 2016; Vaneechoutte et al., 2017). Notably, genes encoding alternatively spliced transcripts are not necessarily differentially expressed during developmental transitions, suggesting that AS shapes the transcriptome independently from transcriptional regulation (Srinivasan et al., 2016). These findings are confirmed by deep-sequencing studies tracking expression and AS changes during the first stages of plant development (Aghamirzaie et al., 2013; Lu et al., 2013; Sun and Xiao, 2015; Qu et al., 2016; Thatcher et al., 2016; Narsai et al., 2017). The detection of prominent AS switches and of development-specific splice variants corroborates an important regulatory layer of early plant development at the splicing level. Interestingly, RNA-processing factors themselves undergo AS resulting in a potential autoregulatory feedback loop.

During embryogenesis in soybean (Aghamirzaie et al., 2013), AS of 47,331 genes produced 217,371 different transcripts, most of which had not been previously identified. Nearly one third of the genes showed variations in transcript levels during embryo development, including those encoding enzymes involved in carbon or nitrogen metabolism and hormone-mediated signaling pathways. Most AS events were detected during the later stages of embryogenesis, i.e., embryo maturation, dehydration, establishment of dormancy, and at the quiescent state. This induction of AS may be explained by the striking clustering of both splicing-related and ABA-associated factors observed at the late phases of seed development. Seed maturation and desiccation, which involve very specific developmental, hormonal, and biochemical processes, were also examined in arabidopsis (Srinivasan et al., 2016), where RNA-seq profiling was performed on developing and mature seeds. Interestingly, transcription and AS showed opposite trends, with transcription declining during seed maturation, while AS increased. Over a quarter of the loci undergoing AS expressed stage-specific splice variants or showed a marked isoform switch, with a striking 88% of the detected AS events being absent from the TAIR10 genome annotation. Again, there were no significant changes in total transcript levels of many alternatively spliced genes, pointing to AS as an important regulatory mechanism operating independently from transcription. Most of the genes exhibiting differential splicing were involved in RNA processing, potentially amplifying the AS regulatory effect in preparation for seed germination.

Two recent studies addressed the AS contribution during seed germination. In barley embryos, 14–20% of multiexon genes expressed multiple mRNA isoforms, some of which displayed clear changes during early germination (Zhang et al., 2016). Surprisingly, the most prominent AS event was alternative 3′SS selection, and there were no substantial alterations in total transcript levels for most genes. Assessment of the biological functions of the genes undergoing AS during germination indicated involvement in protein synthesis, energy and carbon metabolism as well as RNA transport and splicing. Overall, seed germination appears to require expression of a specific set of genes, with AS playing a widespread role. The regulatory potential of AS during germination is underscored by a subsequent report in arabidopsis (Narsai et al., 2017) confirming the expression of time- and tissue-specific mRNA variants, the occurrence of dynamic changes in isoform abundance, and that splicing regulators are major AS targets during this developmental process.

AS regulation during early plant growth is also relevant in the context of environmental responses. Light, which is perceived by various photoreceptors, strongly impacts the life cycle of plants, regulating among others early developmental steps such as seed germination and the transition to autotrophic growth. Genome-wide effects of light on plant AS were recently analyzed by RNA-seq (Wu et al., 2014; Mancini et al., 2016), including in very young seedlings (Shikata et al., 2014; Hartmann et al., 2016). Shikata et al. (2014) reported that, during the initial response of etiolated seedlings to red light, the number of genes showing phytochrome-mediated differential gene expression or changed AS pattern is comparable, while later transcription becomes the dominant regulatory mechanism. In the phytochrome-dependent AS dataset, splicing-related genes were overrepresented, including SR proteins and the U1 and U2 spliceosomal subunits, while transcription factors comprised the major group of differentially expressed genes. AS seemed to play a significant role in light-induced chloroplast differentiation, as photosynthesis- and plastid-related genes were also enriched in the differential AS sets. When Hartmann et al. (2016) analyzed the response of etiolated arabidopsis seedlings exposed blue, red, or white light treatments, ∼20% of genes were found to be differentially expressed, with ∼700 AS events being detected, most of which mapped to coding sequences. Again, gene ontology analysis revealed overrepresentation of the RNA-binding category, including many splicing factors. A link between light-induced AS and mRNA stability was also uncovered, with 77.2% of the detected mRNA isoforms more abundant in the dark samples being potential NMD targets. Remarkably, in most of AS events, an isoform switch from a putative instable mRNA variant to a protein-coding alternative occurred upon light exposure. Moreover, mutants lacking the major red or blue light receptors showed impaired AS mainly when subjected to monochromatic red or blue light, indicating that additional signaling pathways influence AS under white light. The authors suggested that metabolic signals, sugars in particular, are implicated in light-mediated AS regulation.

## Splicing Factors Regulating Early Plant Development

Compelling evidence from large-scale analyses pointing to an important role for AS during early plant development is being substantiated by accumulating *in vivo* genetic studies (**Table 1**). Overexpression or complete abrogation of splicing function often causes embryo lethality, indicating that the corresponding genes are essential for viability and development of a functional plant (Kalyna et al., 2003; Schmitz-Linneweber et al., 2006; Liu et al., 2009; Kim et al., 2010; Fouquet et al., 2011; Swaraz et al., 2011; Perea-Resa et al., 2012; Shikata et al., 2012; Sasaki et al., 2015; Tsugeki et al., 2015). Some studies have established a hormonal basis for the embryo and early seedling development defects caused by altered expression of splicing factors (Kalyna et al., 2003; Casson et al., 2009; Tsugeki et al., 2015), with abnormal spatial distribution of auxin arising from erroneous splicing and expression of auxin biosynthesis, transport, and signaling genes. A link between mRNA splicing and auxin signaling was also uncovered in flowers, where subcellular compartmentation of an auxin biosynthetic gene is regulated by AS (Kriechbaumer et al., 2012).

**Table 1:** Splicing factors and targets functioning in early plant development.

	Gene	Organism	Molecular function	Biological process	Reference
**RNA splicing factors involved in early development**					
	*ABO5*	Arabidopsis	Mitochondrion-targeted PPR protein	Early seedling development	Liu et al., 2010
	*AEF1*	Arabidopsis	Plastid-targeted PPR protein	Early seedling development	Yap et al., 2015
	*CUV*	Arabidopsis	DEAH-box RNA-dependent ATPase homolog	Embryogenesis, early seedling development	Tsugeki et al., 2015
	*Dek35*	Maize	Mitochondrion-targeted PPR protein	Seed development	Chen et al., 2017
	*EMP4*	Maize	Mitochondrion-targeted PPR protein	Seed development, seedling growth	Gutierrez-Marcos et al., 2007
	*ESP1*	Arabidopsis	RNA 3′end processing factor	Seed dormancy	Cyrek et al., 2016
	*FY*	Arabidopsis	RNA 3′end processing factor	Seed dormancy	Jiang et al., 2012; Cyrek et al., 2016
	*GFA1*	Arabidopsis	U5 snRNP component (spliceosomal protein)	Embryogenesis	Liu et al., 2009
	*LSM8*	Arabidopsis	U6 snRNP component (Sm-like protein)	Seed and early seedling development	Perea-Resa et al., 2012
	*MDF*	Arabidopsis	RS domain protein	Early seedling development	Casson et al., 2009
	*NTR1*	Arabidopsis	Spliceosome disassembly factor	Seed dormancy	Dolata et al., 2015
	*PCSF4*	Arabidopsis	RNA 3′end processing factor	Seed dormancy	Cyrek et al., 2016
	*PPR4*	Arabidopsis	Chloroplast-targeted PPR protein	Embryogenesis	Schmitz-Linneweber et al., 2006
	*PPR4*	Maize	Chloroplast-targeted PPR protein	Seedling growth	Schmitz-Linneweber et al., 2006
	*PRMT4*	Arabidopsis	Protein arginine methyltransferase	Hypocotyl elongation in response to light	Hernando et al., 2015
	*PRMT5*	Arabidopsis	Protein arginine methyltransferase	Hypocotyl elongation in response to light	Hernando et al., 2015
	*PRP8*	Arabidopsis	Core spliceosomal protein	Embryogenesis	Sasaki et al., 2015
	*RGH3*	Maize	U2AF35-related (spliceosomal protein)	Embryo, endosperm and seedling development	Fouquet et al., 2011
	*RRC1*	Arabidopsis	RS domain (SR-like) protein	Embryogenesis, early seedling development, hypocotyl elongation in response to light	Shikata et al., 2012
	*RSZ33*	Arabidopsis	SR-protein splicing factor	Embryogenesis, early seedling development	Kalyna et al., 2003
	*RTF2*	Arabidopsis	Rtf2-domain splicing-related protein	Embryogenesis	Sasaki et al., 2015
	*SAD1/LSM5*	Arabidopsis	U6 snRNP component (Sm-like protein)	Seed dormancy	Xiong et al., 2001
	*SFPS*	Arabidopsis	U2-associated splicing factor	Hypocotyl elongation in response to light	Xin et al., 2017
	*SLO3*	Arabidopsis	Mitochondrion-targeted PPR protein	Seed development, germination, early seedling development	Hsieh et al., 2015
	*SmD3-a SmD3-b*	Arabidopsis	snRNP core subunits (spliceosomal proteins)	Embryogenesis, early seedling development	Swaraz et al., 2011
	*SUA*	Arabidopsis	RNA-binding protein	Seed dormancy	Sugliani et al., 2010
	*U11/U12-31K*	Arabidopsis	U12-type spliceosomal protein	Embryogenesis	Kim et al., 2010
**Functional AS targets in early development**					
	*ABI3*	Arabidopsis	B3 domain-containing transcription factor	Seed maturation and dormancy	Sugliani et al., 2010
	*ABI3*	Pea	B3 domain-containing transcription factor	Seed maturation and dormancy	Gagete et al., 2009
	*ABI3*	Linseed flax	B3 domain-containing transcription factor	Seed maturation and dormancy	Wang et al., 2018
	*ABI3*	Tomato	B3 domain-containing transcription factor	Seed maturation and dormancy	Gao et al., 2013
	*ABI5*	Rice	bZIP transcription factor	Seed maturation and dormancy	Zou et al., 2007
	*COP1*	Arabidopsis	E3 ubiquitin protein ligase	Skotomorphogenesis	Zhou et al., 1998
	*DOG1*	Arabidopsis	Unknown	Seed dormancy	Bentsink et al., 2006; Nakabayashi et al., 2015
	*HPR*	Pumpkin	Hydroxypyruvate reductase	Early seedling development	Mano et al., 1999
	*HYH*	Arabidopsis	bZIP transcription factor	Hypocotyl elongation in response to light	Sibout et al., 2006; Li et al., 2017
	*PIF6*	Arabidopsis	bHLH transcription factor	Seed dormancy, seed germination, hypocotyl elongation in response to light	Penfield et al., 2010
	*SPA3*	Arabidopsis	WD40 protein with kinase domain	Hypocotyl elongation in response to light	Shikata et al., 2014
	*SR30*	Arabidopsis	SR protein splicing factor	Light-regulated AS during photomorphogenesis	Hartmann et al., 2016, 2018
	*VP1*	Rice	B3 domain-containing transcription factor	Seed maturation and dormancy	Fan et al., 2007
	*Vp1*	Wheat	B3 domain-containing transcription factor	Seed maturation and dormancy	McKibbin et al., 2002; Wilkinson et al., 2005

Seed dormancy and germination are also strongly affected in mRNA processing mutants. These effects were mostly reported to relate to splicing (Dolata et al., 2015) and polyadenylation (Cyrek et al., 2016) of the *DOG1* gene, a key seed dormancy regulator and known AS target, and to changes in ABA signaling (Xiong et al., 2001; Sugliani et al., 2010; Jiang et al., 2012). Early seedling development can be affected as a manifestation of wider pleiotropic defects (Liu et al., 2010; Swaraz et al., 2011; Perea-Resa et al., 2012; Shikata et al., 2012; Hsieh et al., 2015; Yap et al., 2015) or in weak alleles of embryo lethal mutants (Kalyna et al., 2003; Gutierrez-Marcos et al., 2007; Fouquet et al., 2011; Tsugeki et al., 2015). Observed phenotypes include disturbed cotyledons, hypocotyls, vasculature patterning, roots and/or seedling viability and growth. Notably, mRNA splicing in plastids and mitochondria appears to be crucial for seed development and plant growth in both arabidopsis and maize (Schmitz-Linneweber et al., 2006; Gutierrez-Marcos et al., 2007; Liu et al., 2010; Hernando et al., 2015; Hsieh et al., 2015; Yap et al., 2015; Chen et al., 2017).

Genetic and molecular analyses have confirmed a role for splicing factors in photomorphogenesis, particularly in red-light responses. Phytochrome-dependent light signaling influences AS through specific splicing components, with additional splicing factors such as SR proteins being differentially processed in loss-of-function mutants of these effectors under various light conditions (Shikata et al., 2012; Hernando et al., 2015; Xin et al., 2017). Interestingly, Xin et al. (2017) demonstrated red light-dependent direct interaction and colocalization of a splicing factor and phytochrome B.

## Alternative Splicing Targets Affecting Early Plant Development

Despite massive transcriptome changes imposed by AS during early plant development, only a handful of alternatively spliced transcripts have had their functional significance analyzed in detail (**Table 1**). While one group, including the arabidopsis *DOG1* gene as well as the *OsABI5* and *ABI3/VP1* transcription factors, plays roles in seed maturation, dormancy, and ABA responses (McKibbin et al., 2002; Wilkinson et al., 2005; Bentsink et al., 2006; Fan et al., 2007; Zou et al., 2007; Gagete et al., 2009; Sugliani et al., 2010; Gao et al., 2013; Nakabayashi et al., 2015; Wang et al., 2018), another is important for light signaling and includes *COP1*, *HYH*, and *SPA3* (Zhou et al., 1998; Sibout et al., 2006; Shikata et al., 2014; Li et al., 2017). Moreover, *PIF6* regulates both seed dormancy and light responses (Penfield et al., 2010). Despite the few individual events studied, AS is known to act via diverse mechanisms, as illustrated below.

In agreement with results from large-scale studies, the expression of numerous individual mRNA variants was found to be development- or tissue-specific (Zhou et al., 1998; Fan et al., 2007; Gagete et al., 2009; Sugliani et al., 2010; Gao et al., 2013; Wang et al., 2018), with some turning out to be non-functional, either because they did not produce an active protein or no phenotypic consequence was observed as a result of ectopic expression (Wang et al., 2018). In another study, genetic complementation tests indicated that the different splice variants perform functions equivalent to the constitutive form, even when lacking crucial amino acid sequences or domains (Li et al., 2017). Similarly, AS did not fundamentally influence DNA-binding or protein-protein interaction ability of the ABI3 and ABI5 transcription factors from different plant species, though the binding strength appeared to differ among the various isoforms (Zou et al., 2007; Gagete et al., 2009; Gao et al., 2013).

Alternative splice variants can also fulfill similar or distinct functions depending on developmental stage. The constitutive and alternative PIF6 mRNA variants similarly influenced light responses in seedlings, while only the short isoform displayed evident functions during seed germination (Penfield et al., 2010). In the case of *COP1* and *SPA3*, ectopic overexpression of alternative splice forms phenocopied knock-out mutant phenotypes, indicating that some alternative forms can interfere with the function of the full-length protein (Zhou et al., 1998; Shikata et al., 2014). Strikingly, co-expression and direct protein interactions were found to be necessary for full DOG1 function (Nakabayashi et al., 2015). In genetic complementation assays, independent expression of individual *DOG1* isoforms driven by the native promoter did not restore seed dormancy, whereas transgenic lines carrying two or more *DOG1* variants showed improved dormancy. Detailed analysis of these results supported the hypothesis that, although single isoforms are active, the presence of multiple isoforms is required for adequate DOG1 function. On the other hand, AS-induced changes in protein sequence may lead not only to diminished biological function but, as demonstrated for an *HYH* isoform lacking a protein interaction domain for proteasomal degradation, also to a more stable and hence more active protein isoform (Sibout et al., 2006).

Subcellular targeting provides specialized locations for intracellular processes and can interfere with the regulatory and biochemical potential of proteins. AS of a pumpkin hydroxypyruvate reductase (HPR) acting in photorespiration affected the C-terminal targeting sequence, with one splice form localizing in the peroxisome and another in the cytosol (Mano et al., 1999). The two mRNAs were expressed at similar levels in darkness, while light promoted the production of the shorter, cytosol-localized variant. Most recently, retention of an mRNA variant of the arabidopsis SR30 splicing regulator in the nucleus was shown to influence mRNA stability by preventing the degradation of a potential NMD target in the cytoplasm and its association to the translation machinery (Hartmann et al., 2018).

## Conclusion and Perspectives

Recent transcriptome-wide, genetic and molecular studies have demonstrated that regulation of the complex developmental steps from embryogenesis to establishment of a functional plant includes posttranscriptional control via AS. Seed maturation, establishment and maintenance of seed dormancy, and young seedling responses to light stand out as significant AS-regulated processes. The detection of time- and tissue-specific mRNA variants and of notable switches in splicing patterns substantiate crucial roles for AS in other early development processes. Further large-scale analyses in different tissue types using the latest sequencing technologies and single-cell approaches will be key to understand the full extent of AS events occurring during the initial stages of plant development. Improved standardization of data processing and analysis along with more meticulous experimental set-ups should also allow for more reliable comparative studies. Comprehensive publicly available databases, providing a detailed and up-to-date view of AS in plants are still lacking. These will be pivotal in pinpointing promising novel splice forms and assist in functional studies to distinguish biologically relevant AS contributing to proteomic diversity or gene expression regulation from non-functional AS events and splicing noise. Importantly, state-of-the-art methodology such as iCLIP is proving successful in plant systems and should allow identification of the mRNAs targeted directly by splicing factors to control early plant development.

## Author Contributions

All authors listed have made a substantial, direct and intellectual contribution to the work, and approved it for publication.

## Conflict of Interest Statement

The authors declare that the research was conducted in the absence of any commercial or financial relationships that could be construed as a potential conflict of interest.
